# Chemical and Biological Profile and Allergenicity of *Thymus baicalensis* Plant of Mongolian Origin

**DOI:** 10.3390/antiox10121905

**Published:** 2021-11-28

**Authors:** Tuya Narangerel, Michał Sójka, Radosław Bonikowski, Konrad Jastrząbek, Witold Sroczyński, Aleksandra Plucińska, Alina Kunicka-Styczyńska, Krzysztof Śmigielski, Iwona Majak, Adrian Bartos, Joanna Leszczyńska

**Affiliations:** 1 Institute of Natural Products and Cosmetics, Lodz University of Technology, Stefanowskiego 2/22, 90-537 Lodz, Poland; tuya.narangerel@p.lodz.pl (T.N.); radoslaw.bonikowski@p.lodz.pl (R.B.); konrad.jastrzabek@p.lodz.pl (K.J.); witold.sroczynski@p.lodz.pl (W.S.); 2 Institute of Food Technology and Analysis, Lodz University of Technology, Stefanowskiego 2/22, 90-537 Lodz, Poland; michal.sojka@p.lodz.pl (M.S.); iwona.majak@p.lodz.pl (I.M.); 3 Institute of Fermentation Technology and Microbiology, Lodz University of Technology, Wolczanska 171/173, 90-924 Lodz, Poland; aleksandra.plucinska@dokt.p.lodz.pl (A.P.); alina.kunicka@p.lodz.pl (A.K.-S.); 4 Department of Environmental Biotechnology, Lodz University of Technology, Wolczanska 171/173, 90-924 Lodz, Poland; krzysztof.smigielski@p.lodz.pl; 5 Department of Bioinorganic Chemistry, Medical University of Lodz, Muszynskiego 1, 90-151 Lodz, Poland; adrian.bartos@umed.lodz.pl

**Keywords:** allergy, antimicrobial, antioxidant, essential oils, lipids, medicinal plant, polyphenols, Mongolia, *Thymus baicalensis*

## Abstract

*Thymus baicalensis* is a medicinal plant recognized as a traditional Mongolian therapeutic and health-promoting food supplement. The aim of the study was to check the suitability of the tested plant for supporting the treatment of certain diseases. The following study is the first one to showcase the versatile scope of characteristics of *T. baicalensis*, including its volatile oil composition, polyphenolic composition, lipid composition, phenolic and flavonoid contents, antioxidant activity, antimicrobial properties and ingestive allergenicity. Myrcene, at 26.15%, was shown to be the most abundant component of the volatile oil. Compounds known as inherent components of the *Thymus* genus: thymol and carvacrol made up only about 0.24% of the extracted oil. As much as 10.11 g kg^−1^ of polyphenol compounds were identified as derivatives of luteolin-7-O-glucuronide. The lipid extract was found to be rich in palmitic acid (31.05%), while unsaturated fatty acids were not reported. Spectrophotometric determination of the phenols and flavonoids indicated 7.541 mg of gallic acid g^−1^ and 4.345 mg of quercitin g^−1^, respectively. The free radical scavenging activity was determined by the 2,2-difenylo-1-pikrylohydrazyl method at IC_50_ = 206.97 µg mL^−1^. The extracts also had a strong inhibitory effect on *M. flavus* and *P. fluorescenes* bacteria, as well as *S. cerevisiae* yeasts. The Bet v 1 and profilin allergens in *T. baicalensis* were reported at 175.17 ng g^−1^ and 1.66 ng g^−1^, respectively.

## 1. Introduction

Genus *Thymus* L. of the *Lamiaceae* family (*Nepetoideae* subfamily) comprises about 215 species of aromatic perennial herbs and subshrubs, which occur in Europe, North Africa, Asia and the Mediterranean region [1]. Baikal thyme (*T. baicalensis*) is broadly distributed in the Siberian area and Mongolian territory [2]. The species is understudied and poorly described in the scientific literature [3].

Existing data on the properties of *Thymus* imply the occurrence of pharmacologically and therapeutically significant compounds inherent to the plant species. This includes phenolic acids (ferulic, caffeic, *p*-coumaric, rosmarinic and chlorogenic); flavonoids (quercetin, rutin, catechin, apigenin and luteolin) and their derivatives, as well as trace elements (iron, copper, zinc and manganese) [4,5,6,7]. Some of these compounds, with their pronounced bioactive characteristics, confer antioxidant properties to plant-based orally administered products, like foods and natural medicines. Dietary antioxidants effectively delay oxidative degradation caused by free radicals and counteract various pathologies [8]. According to the scientific literature, polyphenolic compounds of *Thymus* can help prevent a number of chronic diseases, such as neurological, cardiovascular disorders, Parkinson’s and Alzheimer’s diseases and diabetes. In addition, they can improve memory and cognitive functions, as well as provide gastrointestinal protection. These natural components also possess potential biological activities by exhibiting anti-inflammatory, analgesic, antineoplastic, antiosteoporotic and antimicrobial properties [9,10,11].

The literature on the composition and action of essential oils obtained from herbal plants is quite rich [12]. The results of the research on oils obtained from the genus *Thymus* [13,14] from the Buryatia region (Russia) were also described, showing a fairly large diversity of their composition. However, the essential oil of *T. baicalensis* from Mongolia has not been studied.

Plants can be an important source of lipids, especially essential unsaturated fatty acids (EFAs) [15,16]. Herbs can supplement our diet, especially in deficient ω-3 fatty acids. Some fatty acids are precursors of bioactive compounds, including those having antimicrobial activity [17]. *Thymus baicalensis* is used in traditional medicine in Mongolia (Table 1).

The research should verify the hypothesis that the studied herbs may support the treatment of selected diseases.

Given the potential pharmacognostic application of *Thymus*, the present study was performed to determine the chemical compositions of the volatile oils, phenolic compounds and lipids extracted from *T. baicalensis* and to test their antioxidant, antimicrobial and allergenic effects. The findings of this article are a valid contribution to broaden our ethnobotanical knowledge on an obscure *Thymus baicalensis* plant species.

## 2. Materials and Methods

### 2.1. Sample Collection

Medicinal plant *T. baicalensis* was obtained from the local market in Ulaanbaatar, Mongolia. Collection took place in Ulaan-Uul sum, Khuvsgul Province in August 2016. Botanical identification of the plant was confirmed by Dr. Urgamal Magsar from the Institute of General and Experimental Biology (Mongolian Academy of Sciences). Plant material was subjected to drying in the shade at 15–17 °C for three weeks. The aerial part of the plant was gently ground using a pestle and mortar and packed in PE plastic ziplock bags. They were stored in a dark place at room temperature at all times.

### 2.2. Extraction of Phenolic and Flavonoid Compounds

Weighted 0.500-g portions of finely ground samples were extracted with 12 mL of 50% methanol (*v*/*v*). Methanol was prior acidified with 1% of formic acid (*v*/*v*) and mixed using a shaker (TTS 2, Yellow Line, IKA-Werke, Staufen, Germany) at room temperature for one hour. Acidification was performed to avoid oxidation of the polyphenols. The supernatant was then separated via centrifugation at 10,000 rpm, for 5 min (MPW-251, Med instrument, Warsaw, Poland) and passed through paper filters (BOECO Germany Grade 3 hw). The sediment was reextracted with 10 mL of 50% methanol (*v*/*v*), and the separation process was repeated. Finally, collected supernatants were combined into a 25-mL volumetric flask and filled up to the mark with 50% methanol. They were kept in the freezer (−20 °C) until tested.

### 2.3. Total Phenolic and Flavonoid Content

To determine the total phenolic content, 50 µL of the 50% methanol extract was mixed with 250 µL of Folin–Ciocalteu’s reagent and 2.5 mL of 20% sodium carbonate solution in a 25-mL volumetric flask and filled with deionized water up to the graduation mark. The mixture was incubated for one hour in a dark place. The absorbance of the mixture was measured at 720 nm using spectrophotometer HP8453 (Hewlett Packard/Agilent, Santa Clara, CA, USA). All samples were tested in triplicate. The results were expressed as milligrams of gallic acid per gram of plant dry mass.

Gallic acid 250-µg mL^−1^ standard solution was used to prepare reference dilutions at a concentration range from 5 to 150 µg mL^−1^. The total phenolic content of the samples was estimated using calibration curves predetermined with gallic acid.

For total flavonoid content determination, a 500-µL sample extract was mixed with 1.5 mL of 80% methanol (*v*/*v*), 100 µL of 10% AlCl3, 100 µL of CH3COONa (1 M) and 3.0 mL of deionized water. The incubation was performed in a dark place for 30 min. Absorbance was measured at 415 nm using spectrophotometer HP8453 (Hewlett Packard/Agilent, Santa Clara, CA, USA). All samples were tested in triplicate. The results were expressed as milligrams of quercetin equivalents (QE) per gram of dry plant mass.

For the preparation of the calibration curve, 200 µg mL^−1^ of quercetin was dissolved in 80% methanol (*v*/*v*) and used as the stock solution. Dilutions were prepared at concentrations between 5 and 100 µg mL^−1^. The total flavonoid content was estimated using a calibration curve predetermined with quercetin.

### 2.4. Free Radical Scavenging Activity and Calculation of the IC_50_

First, 0.1 mM of DPPH solution was prepared through dilution in 80% methanol (*v*/*v*). Trolox at a concentration of 80 µg mL^−1^ was used as a standard antioxidant. The DPPH solution at a volume of 2.0 mL was combined with 2.0 mL of the plant extract. The same was applied for the standard solution, adjusted prior to the following concentrations: 5, 10, 20, 30, 40 and 50 µg mL^−1^. The mixture was blended in a vortex and allowed to stand in the dark for 30 min. Absorbance was measured at 517 nm using UV–Vis spectrophotometer HP8453 (Hewlett Packard/Agilent, Santa Clara, CA, USA). In the blank control, the sample was substituted with deionized water. All analyses were performed in triplicate. The DPPH radical scavenging activity was calculated from the following equation:

RSC, % = 100 (A_0_ − A)/A_0_

where:

A—average absorbance of the sample;
A_0_—average absorbance of a control (DPPH).

**Figure 1:**
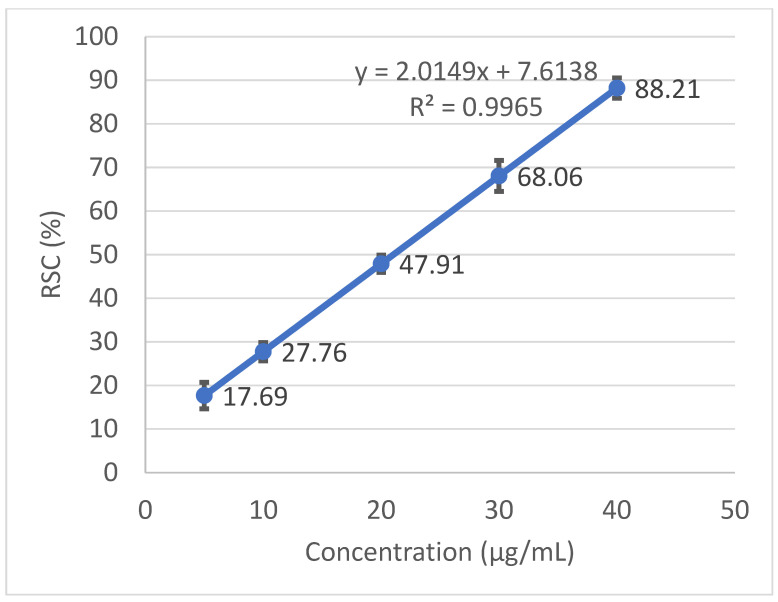
Radical scavenging activity determined in the *T. baicalensis* extract.

**Figure 2:**
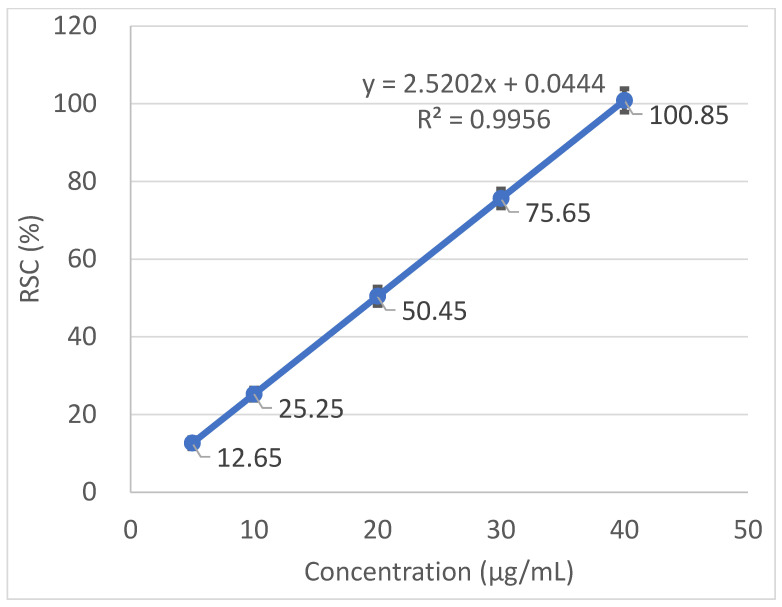
Radical scavenging activity determined in the Trolox antioxidant standard solution.

Radical scavenging capabilities of the *T. baicalensis* extract and Trolox antioxidant standard solution are shown in Figure 1 and Figure 2, respectively.

The inhibitory concentration was plotted against the free radical scavenging activity measured in the samples. The radical scavenging activity of the methanol extract samples was calculated using the linear (*y* = a*x* + b) equation plotted with the DPPH values, with the *Y*-value being 50 and the *X*-point being the IC_50_ value. The value was expressed as micrograms of Trolox equivalents per mL of methanol extract.

### 2.5. GC-MS Determination of Volatile Oil Components

Essential oils were isolated through hydrodistillation from 5.00-g dry plant-weighted portions using a Clevenger-type apparatus. The makeup of volatile compounds in the oil composition was determined using Thermo Trace GC Ultra/DSQ II (Thermo Fisher Scientific, Waltham, MA, USA). Operating parameters of the gas chromatography were set up as follows: column: nonpolar stationary phase Rxi^−1^ ms (length 60 m, internal diameter 0.25 mm and, film thickness 0.25 μm; Restek Corp., Bellefonte, PA, USA); injector temperature: 280 °C; FID detector temperature: 300 °C; carrier gas: helium, constant pressure 300 kPa and split ratio 1:100; oven temperature program was 50–300 °C at 4°/min. Mass spectrometry parameters: ion source temperature 200 °C and ionization energy 70 eV. The quantity of the individual components was expressed as a percentage of the essential oil and was achieved using a flame ionization detector connected through the MS-FID splitter (SGE Analytical Science, Ringwood, Melbourne, VIC, Australia). Databases from the NIST Library, Wiley 8th edition and the Adams 4th edition were used.

### 2.6. LC-MS Determination of Phenolic Compounds

Identification and quantification of phenolic compounds in methanol extracts was carried out using a Dionex Ultimate 3000 HPLC coupled with a DAD and Q Exactive Orbitrap mass spectrometer (Thermo Fisher Scientific, Waltham, MA, USA).

The operating parameters of the LC-MS system [18] were set up as follows: column—250 mm × 4.6 mm, 5 µm, Luna C18(2) 100 Å, column temperature: 35 °C, mobile phase flow rate 1 mL min^−1^ and injection volume 20 µL. The mobile phases consisted of 1% formic acid (*v*/*v*) in water (solvent A) and 80:20 (*v*/*v*) acetonitrile:water (solvent B). The following gradient was used: 0–6.5 min, 5% (*v*/*v*) B; 6.5–12.5 min, 5–15% (*v*/*v*) B; 12.5–44 min, 15–45% (*v*/*v*) B; 44–45 min, 45–75% (*v*/*v*) B; 45–50 min, 75% (*v*/*v*) B; 50–52 min, 75–5% (*v*/*v*) B and 52–65 min, 5% (*v*/*v*) B.

The MS system coupled to HPLC was an Orbitrap mass spectrometer equipped with an H-ESI probe used in the negative mode. The mass detector parameters were set up as follows: vaporizer temperature 500 °C, ion spray voltage: 4 kV, capillary temperature: 400 °C, sheath gas: 75 units, auxiliary gas: 20 units and scan ranged from 200 to 2000 *m*/*z*.

The detector was operated in either full MS or full MS/dd-MS2 scan modes. To generate MS2 data, the full MS/dd-MS2 scan mode was applied. The collision energy used to generate MS2 spectra was set to 20. Tuning and optimization were performed using the direct injection of extract diluted in an 80:20 (*v*/*v*) mixture of mobile phases A and B at a flow rate of 0.25 mL min^−1^. Chromatographic data of the *T. baicalensis* extracts was collected using Xcalibur software (Thermo Fisher Scientific).

The contents of the polyphenolic compounds determined by LC analysis in methanol extracts of *T. baicalensis* were calculated using standard curves plotted with the use of chlorogenic acid (purity > 95%, Sigma-Aldrich, Steinheim, Germany) at 320 nm for compounds number 2, 3 and 6 and at 280 nm for compound 1 (Table 2); rosmarinic acid (purity > 98%, Sigma-Aldrich) at 280 nm for compounds 5 and 10 (Table 2) and kaemferol-3-rutinoside (purity 98%, Extrasynthese, Genay, France) at 360 nm for compounds 4, 7, 8 and 9 (Table 2). 

### 2.7. Extraction of Lipids

To obtain lipid extracts, weighted portions of 15-g dry plant were subjected to extraction with isooctane. Afterwards, the solvent was slowly vaporized using a rotary vacuum evaporator. The residue (lipid extract) from the distillation flask was collected into a glass test tube. The extract was stored at 4 °C.

### 2.8. GC-MS Determination of Lipids

The amount of 10.0 mg of the extracted sample was mixed with 100 µL of methyl nonadecanoate solution, which was used as the internal standard in anhydrous tert-butyl methyl ether (0.2 mg/mL) and 100 µL of trimethylsulfonium hydroxide solution ~0.25 M in methanol. The solution was incubated at 80 °C for 30 min, and 1 µL of the output mixture was subjected to GC-MS analysis. The GC-MS determination was performed with a gas chromatograph coupled with a time-of-flight mass spectrometry detector (Pegasus 4D, LECO, St. Joseph, MI, USA).

The operating parameters were set up as follows: Stabilwax-DA capillary column (length 20 m, internal diameter 0.18 mm and 0.18-µm film thickness; Restek Corp., Bellefonte, PA, USA). The oven temperature program was 50–245 °C at 4° min^−1^, carrier gas helium and flow rate 1 mL min^−1^. Identification of the lipids was based on the comparison of their mass spectra with data available from the commercial database, and the retention times of the methyl esters were compared with the standards (37 Component FAME Mix, Supelco, St. Louis, MO, USA; Cat. No. CRM47885). The contents of the individual components were expressed as a percentage of the total extracted lipids.

### 2.9. Antimicrobial Activity

A weighted portion of 0.25-g dry plant was extracted with 1.00 mL of 95% ethanol (*v*/*v*) using a shaker (TTS 2, Yellow Line, IKA-Werke, Staufen, Germany) at room temperature for 24 h. The supernatant was passed through filter paper (BOECO Germany Grade 3 hw). Ethanol was evaporated gradually, and the residue was collected.

The following microorganisms were used in the study: bacteria: *Bacillus subtilis* ATCC 6633, *Staphylococcus epidermidis* ATCC 12228, *Staphylococcus aureus* ATCC 6538, *Micrococcus flavus* LOCK 0849, *Pseudomonas aeruginosa* ATCC 45442, *Pseudomonas fluorescens* PCM 2123, *Escherichia coli* ATCC 8739 and *Enterobacter aerogenes* ATCC 13048, as well as fungi: *Candida vini* LOCK 0008, *Saccharomyces cerevisiae* LOCK 0119, *Aspergillus niger* LOCK 0440 and *Penicillium expansum* LOCK 0535. The strains originated from the American Type Culture Collection (ATCC), Polish Collection of Microorganisms Institute of Immunology and Experimental Therapy Polish Academy of Sciences (PCM) and Collection of Pure Cultures of the Institute of Fermentation Technology and Microbiology Technical University of Lodz ŁOCK 105 (LOCK). The microorganisms were activated through double passaging: bacteria on trypticase soy agar medium (TSA for 37 °C, 48 h; Oxoid, Basingstoke, UK), yeast and moulds on Sabouraud dextrose agar medium (SDA for 28 °C, 72-h yeast and 7-day moulds; bioMerieux, Warsaw, Poland).

The test for antimicrobial activity of the extracts was conducted in 96-well microtiter plates by the microdilution method according to CLSI recommendations (CLSI 2015) [19]. One hundred microliters of pure extract were diluted with 100 μL of TSB medium (Trypticase Soy Broth, Merck, Darmstadt, Germany), and then, 100 μL of microorganism suspension was added. Previously prepared suspensions of the tested bacteria and yeast were in a physiological salt solution (0.85% NaCl); the mould was in a physiological salt solution with the addition of 0.5-g L^−1^ polysorbate 80 R and standardized to the density of 10^6^ CFU mL^−1^. Next, 2-fold subsequent dilutions of the extract were made. The extract concentrations tested were in the range of 33.333–0.130% (*v*/*v*). The following negative controls were conducted: the pure extracts without microorganisms and the culture with suspension of one microorganism only. The positive controls were the bacteria and fungi cultures with the addition of novobiocin (0.5 μg mL^−1^) and cycloheximide (0.2 μg mL^−1^), respectively.

All plates were incubated in 30 °C for 24 h for bacteria and in 28 °C for 48 h for fungi. After incubation, the results of the experiment were examined with the use of 0.01% solution of resazurin. Ten microliters of the solution were added to each well on the plate and then incubated for 2 h in 30 °C. The viability of the microorganisms was estimated macroscopically, assuming that the red color of the well indicates the presence of living organisms, while the blue color stands for a lack of living microorganisms. On the basis of color changes, the MIC values of the extracts were determined as the lowest extract concentrations inhibiting the growth of the microorganisms. The experiments were conducted in triplicate for each microorganism tested.

### 2.10. Extraction of Food Allergens

Weighted portions of 0.100-g plant materials were ground against and pressed through a filter 60 times using a plastic rod with twisting force. Then, 1000 µL of the native lysis buffer was added, and the sample was ground 60 times more with twisting force. The filter cartridge was transferred to the refrigerator for 12 h. Afterwards, the supernatant was subjected to centrifugation at 10,000 rpm (MPW-251, Med instrument, Warsaw, Poland) for 3 min and deposited in the freezer (−20 °C).

### 2.11. Allergenic Protein Content Determination

Allergenicity of the extracts was determined with the ELISA method. The volume of 100 μL of the supernatant was used to coat 96-well microplates (SPL Lifesciences, Gyeonggi-do, South Korea) and was left for overnight incubation at 4 °C. On the next day, the microplates were washed with 3 × 350 μL PBS–T washing buffer (pH = 7.4 PBS, 0.1% Tween-20). Afterwards, 3% low-fat milk in PBS–T washing buffer was used to block unbound sites in the wells through 2-h incubation at room temperature. The milk solution was removed and washed with 3 × 350 μL of PBS–T washing buffer. A volume of 100 µL of diluted (1:1000) primary rabbit anti-profilin antibody or diluted (1:500) mouse anti-Bet v 1 antibody was applied and incubated at room temperature for one or two hours, respectively. After incubation, the washing cycle was repeated, and 100 µL of diluted (1:5000) anti-rabbit secondary antibody or anti-mouse IgG conjugated to alkaline phosphatase was added, followed by one hour of incubation at room temperature. The microplate was washed with 3 × 350 μL of PBS–T washing buffer. It was then incubated with pNPP at 100 µL applied per well for one hour at room temperature. The reaction was stopped with 50 µL of 3-M NaOH. A multiscan RC reader (ThermoLabsystem, Helsinki, Finland) was used to measure the absorbance at 405 nm. The allergen protein values were expressed as nanograms per gram of the sample.

Calibration curves were plotted for Bet v 1 and profilin. As for Bet v 1, the curve was prepared with standard solutions at concentrations between 0.5 and 50 ng mL^−1^ and was shown to follow equation formula *y* = 0.0242*x* − 0.0697 (*R*^2^ = 0.992). For profilin, the curve was prepared with standard solutions at concentrations ranging from 0.5 to 100 ng mL^−1^, and it was shown to follow the formula *y* = 0.0017*x* + 0.07415 (*R*^2^ = 0.994). The content of Bet v 1 and profilin allergen proteins in analytical samples were calculated using standard calibration curves.

## 3. Results

### 3.1. Spectrophotometric Determination of Total Phenolic and Flavonoid Content

Phenols are bioactive plant compounds with an aromatic ring bearing one or more hydroxyl groups. The total phenolic content was determined in *T. baicalensis* at 7.541-mg GAE g^−1^ (milligrams of gallic acid equivalents per gram of plant dry mass). Flavonoids are considered the largest group of naturally occurring phenolic compounds. The total flavonoid content in *T. baicalensis* was found to be 4.345-mg QE g^−1^ (milligrams of quercetin equivalents per gram of plant dry mass).

### 3.2. Antioxidant Activity

A strong antioxidant activity is associated with a high amount of total phenolic content. The IC_50_ (the concentration at which the free radical scavenging activity corresponds to 50%) is a measure of the antioxidant potential. It was determined from a graph that was plotted with values obtained with the use of a standard DPPH method. The output IC_50_ equaled 206.97 µg mL^−1^ in *T. baicalensis* extract. For a comparison, Trolox, a standard antioxidant, yielded an IC_50_ value of 19.37 µg mL^−1^.

### 3.3. GC-MS Determination of Volatile Oil Composition

As a result of the GC-MS determination performed on the volatile oil extracted from *T. baicalensis*, thirty-seven chemical components were identified and accounted for 96.33% of the total oil. These compounds are listed in Table 2. As shown, the myrcene content (26.15%) was found to be the most abundant, followed by terpinen-4-ol (17.12%), α-terpineol (7.06%), γ-terpinene (6.98%), borneol (5.65%), 1,8-cineole (4.43%), α-terpinene (3.37%), caryophylle (3.14%), α-pinene (3.07%) and camphene (2.18%).

### 3.4. LC-MS Determination of Polyphenolic Compounds

The identification of polyphenolic compounds was based on comparing their retention times, UV spectra and MS spectra with those of known standard compounds. In the case of standards being unavailable, the experimental spectral data was compared with data acquired from the literature. As shown in Table 3, 10 compounds were determined, all members of the flavonols and phenolic acid derivatives.

Compounds 1 and 3 were identified as quinic acid and caffeic acid based on their retention time and fragmentation ions. Compounds 2 and 10 were characterized as chlorogenic and rosmarinic acids by comparing their mass spectra with those of commercial standards. Moreover, compound 5 ([M-H] at *m*/*z* 377) was identified as a rosmarinic acid derivative that had an additional phenolic hydroxyl group. Fragmentation ions of this compound were the same as these of compound 10. Compound 4 was characterized as apigenin C-hexoside-C-hexoside. It is often reported in other plants of the *Lamiaceae* family. Compound 6 at *m*/*z* 367 was identified as 5-O-feruloylquinic acid. It derives from quinic and ferulic acid. To our knowledge, no previous investigations on *Thymus* species have reported this derivative. The last three compounds (7–9) were identified as glucuronides of quercetin, apigenin and luteolin derivatives, respectively. Loss of the glucuronyl moiety was observed in their MS fragmentation [(M-H) 176].

### 3.5. LC-MS Determination of Lipids

Twenty-two lipid components were identified in the *T. baicalensis* extract. As listed in Table 4, palmitic acid, a saturated fatty acid, was a major component of the sample and accounted for as much as 31.05% of the extracted lipids. Other compounds found included hexacosane (11.54%), nonacosane (9.57%), oleanolic acid (9.72%), ursolic acid (4.24%), nonanedioic acid, dimethyl (3.97%), arachidic acid (3.49%) and lignoceric acid (3.09%). Interestingly enough, unsaturated fatty acids were not identified in the plant extract.

### 3.6. Antimicrobial Properties

The ethanol extract was evaluated for antimicrobial activity against four strains of Gram-positive bacteria (*Bacillus subtilis* ATCC 6633, *Staphylococcus epidermidis* ATCC 12228, *Staphylococcus aureus* ATCC 6538 and *Micrococcus flavus* LOCK 0849) and four strains of Gram-negative bacteria (*Pseudomonas aeruginosa* ATCC 45442, *Pseudomonas fluorescens* PCM 2123, *Escherichia coli* ATCC 8739 and *Enterobacter aerogenes* ATCC 13048), as well as four strains of fungi (*Candida vini* LOCK 0008, *Saccharomyces cerevisiae* LOCK 0119, *Aspergillus niger* LOCK 0440 and *Penicillium expansum* LOCK 0535). The results of the study were expressed as the minimum inhibitory concentration (MIC) and presented in Table 5.

The inhibitory effect of the *T. baicalensis* extract on the Gram-negative bacteria was overall more pronounced than that exerted on the Gram-positive bacteria. The lowest MIC value (4.162%) was observed on Gram-positive strain *M. flavus* and Gram-negative strain *P. fluorescens*. *B. subtilis* and *S. epidermidis* (Gram-positive) displayed the most resistance (33.333%) to the plant extract. Moreover, *S. cerevisiae* yeasts were the most sensitive to the *T. baicalensis* extract, followed by *C. vini*, *A. niger* and *P. expansum*. On the other hand, the growth of *A. niger* and *P. expansum* fungi exhibited the strongest resistance (8.325%) in the study.

### 3.7. Ingestive Allergenicity

Bet v 1 and profilin are major birch pollen allergens that commonly occur in plant-based foods. The determination performed with the ELISA method indicated the contents of the Bet v 1 and profilin allergens in *T. baicalensis* at 175.17 ng g^−1^ and 1.66 ng g^−1^, respectively.

## 4. Discussion

The following study was the first one to showcase the versatile scope of the characteristics of T. baicalensis, including its volatile oil composition, polyphenolic composition, lipid composition, phenolic and flavonoid contents (TPC and TFC), antioxidant activity, antimicrobial properties and ingestive allergenicity. The total phenolic and flavonoid contents determined in *T. baicalensis* seem to be in line with the existing data on various *Thymus* species. Gedikoğlu and colleagues determined TPC at 15.13-mg GAE g^−1^ and TFC at 7.29-mg QE g^−1^ in *T. vulgaris* obtained from Turkey. Moreover, the same authors reported rosmarinic acid as the main compound in this plant [20]. Tohidi et al. reported TPC and TFC at 31.38–70.56-mg TAE g^−1^ (tannic acid equivalents) and 1.89–8.14-mg QE g^−1^, respectively, as determined across 14 *Thymus* species [21]. Dessalegn and colleagues published data on *Thymus schimperi* R. and *Thymus vulgaris* L., reporting TPC at 46.0 and 45.23-mg GAE g^−1^, respectively, and TFC at 14.7 and 10.65-mg QRE g^−1^ (quercetin equivalents), respectively [14].

The variety of antioxidant properties and their corresponding IC_50_ values reported across *Thymus* species are due to the chemical structure of the plant, its geographical origin, characteristics of the extraction method, applied determination assay and other environmental factors. Tohidi and coworkers reported IC_50_ at the order of magnitude similar to ours, i.e., 273.36–693.8 µg mL^−1^ in distinct *Thymus* species obtained from different regions of Iran [21]. Afonso and colleagues, on the other hand, reported DPPH IC_50_ values that spanned from 1.8 to 44.7 μg/mL in *Thymus vulgaris* alone [22].

In the scientific literature, there have been reports of thymol, carvacrol, *p*-cymene and γ-terpinene being the major constituents of *Thymus* species. In our research, these supposedly dominant components (thymol, 0.16% and carvacrol, 0.08% and β-cymene, 1.08% and γ-terpinene, 6.98%) made up a small fraction (8.62%) of the plant sample. On the other hand, acyclic, bicyclic and menthane monoterpenoids, such as myrcene, (E)-beta-ocimene, terpinen-4-ol, α-terpineol and borneol, were found to be prominent components of *T. baicalensis*. Benchabane et al. and Jarić et al. described similar findings in *Thymus*-tested samples. The authors concluded that thymol and carvacrol were not present in tested *Thymus* species cultivated in Lithuania, Estonia and Spain [23,24]. In another study, myrcene was reported in *Thymus serpyllum* L. up to 74.2% of the total determined volatile compounds, depending on the volatile oil extraction technique [25]. This goes to show how many variables impact the yield and chemical composition of essential oils. This includes the species itself; specific sample parts (leaves, flowers, fruit and root); geographical location; cultivation environment; drying methods (sun, shade and oven); distillation process (solvent type and extraction time); distillation methods (hydro, water and steam); plant age and time of harvesting (beginning and end of flowering and fruiting) [12,13,26]. A comparison of the four most popular compounds (thymol, carvacrol, *p*-cymene and γ-terpinene) found in essential oils of *Thymus* species obtained from different parts of the world is presented in Table 6.

Previous studies on *T. vulgaris* L (Italy) by Simeoni and colleagues indicated 17 individual phenolic compounds. The total phenolic content was reported at 59.35 mg g^−1^, with the most abundant component being rosmarinic acid (13.95 mg g^−1^), followed by chicoric acid (8.76 mg g^−1^), ferulic acid (5.38 mg g^−1^), vanillic acid (5.36 mg g^−1^) and *p*-coumaric acid (3.59 mg g^−1^). Quercetin (0.20 mg g^−1^) was the minor component of the analyte [38]. Rosmarinic acid is a known free radical scavenger and confers antiviral properties to medicinal herbs [39]. Raudone et al. showed that the compound was a notable phenolic component of *Thymus*, but its content exhibited a dramatic reduction after the flowering stage and at the end of vegetation [40]. It was also reported in research on *T. citriodorus* by Pereira and colleagues. Scientists have listed rosmarinic acid, luteolin and apigenin-7-β-O-glucuronides as dominant polyphenolic components in this species [41].

As for the lipid composition, the hexane extract of Algerian *T. capitatus* was shown to contain α-linolenic (29.6%), palmitic (16.6%), linoleic (15.1%) and behenic acid (9.6%) [15]. In 2019, Zaïri and colleagues described the fatty acid content in essential oil from a Tunisian plant of the same species. The authors reported the TSFA content at 2.93 g kg^−1^, TMFA at 0.872 g kg^−1^ and TPFA at 0.375 g kg^−1^ [16]. The fatty acids determined in the leaves of the selected *Thymus* genus were identified by Cacan et al. [42]. As reported, the TSFA content was 21.47%, 26.66%, 51.12%, 30.97% and 27.85% in *T. kotschyanus* var. *glabrescens*, *T. kotschyanus* var. *kotschyanus*, *T. hausknechtii*, *T. pubescens* var. *pubescens* and *T. fallax*, respectively. The content of the TUSFA was determined at 71.32%, 64.66%, 39.05%, 59.43% and 62.68%, respectively [42].

In our research, the second, after SFA, major group of compounds in *T. baicalensis* were acyclic alkanes (30.81%). Most notably, this included hexacosane (11.54%) and nonacosane (9.57%). As indicated by the scientific literature, hexacosane exhibits antimicrobial and antibacterial properties [17].

Phenolic and alkaloid compounds underlie antioxidative and antimicrobial properties of plants [43]. Ahmad and his team explored the antimicrobial activity of thymol and carvacrol-rich essential oils in *T. vulgaris* using the MIC approach. The research indicated that an inhibitory effect against *E. coli* ATCC 8739, *M. cattarhalis* ATCC 23246, *S. aureus* ATCC 126000, *E. faecalis* ATCC 29212, *B. cereus* ATCC 11778, *C. albicans* ATCC 10231 and *C. tropicalis* ATCC 201380 yielded values between 0.125 and 1 µg mL^−1^ [44]. Džamić et al. studied the antibacterial and antifungal properties of volatile oil in *T. capitatus* from Libya. The authors determined the MIC for the following microorganisms: Gram-negative (*E. coli*, *P. aeruginosa*, *S. typhimurium* and *Proteus mirabilis* human isolate); Gram-positive (*L. monocytogenes*, *B. cereus* clinical isolate, *M. flavus* and *S. aureus*) and fungi (*A. flavus*, *A. fumigatus* human isolate, *A. niger*, *A. ochraceus*, *Penicillium funiculosum*, *Penicilium ochrochloron*, *Trichoderma viride* and *Candida albicans* human isolate). The antibacterial and antifungal effects of *T. capitatus* were in the range of 1 to 2 µg mL^−1^ and 0.2–1 µg mL^−1^, respectively [45].

Bet v 1 and profilin are popular birch pollen allergens that commonly occur in plant-based foods [46]. The contents of the Bet v 1 and profilin allergen proteins were determined in cumin, fennel, parsley, anise and coriander by Aninowski et al. The authors reported the Bet v 1 contents at 520–1540, 500–1400, 630–980, 550–1150 and 600–860 ng g^−1^, respectively, while the profilin contents were determined at 9.9, 3.75, 3.27, 3.42 and 12.36 ng g^−1^ [47]. These were in line with the order of magnitude reported in *T. baicalensis* but remained lower, suggesting the safety of the test plant. Unfortunately, the literature on herbal allergens is very sparse, and the problem requires further research.

In conclusion, the contents of allergens in the tested plant, compared to other herbal plants, were very low; on the other hand, the high contents of the polyphenolic compounds soothe inflammatory reactions, including allergic ones, hence the suggestion of a very low allergic potential of *T. baicalensis.*

The confirmed presence of many biologically active compounds, especially those with antimicrobial activity, suggests the usefulness and justification for using *Thymus baicalensis* as a treatment adjunct.

## Figures and Tables

**Table 1:** Examples of the use of the herb *Thymus baicalensis* in traditional medicine in Mongolia.

Disease	Way of Preparing Herbs
Respiratory tract Pharyngeal diseases Periodontal infections	Hot water extract
Antiseptic (cleaner or sanitizer)	Oil extract
Stress and depression symptoms Poor general health	Burning
Balancing progesterone hormone	Oil extract
Alcohol addiction treatment	Water extract

**Table 2:** Chemical components of the volatile oil in *T. baicalensis*.

Compound	Total, %	Compound	Total, %
β-Thujene	0.49	Borneol	5.65
α-Pinene	3.07	Terpinen-4-ol	17.12
Camphene	2.18	α-Terpineol	7.06
Sabinene	1.74	Piperitol, cis	0.23
β-Pinene	1.63	Piperitol, trans	0.15
2,3-Dehydro-1,8-cineole	0.09	Bornyl acetate	0.24
β-Myrcene	26.15	Thymol	0.16
α-Phellandrene	0.08	Carvacrol	0.08
α-Terpinene	3.37	Car-3-ene	0.12
β-Cymene	1.08	β-Bourbonene	0.19
1,8-Cineole	4.43	Caryophylle	3.14
Limonene	0.78	Humulene	0.16
(E)-beta-ocimene	0.03	Germacrene D	1.87
γ-Terpinene	6.98	Bicyclogermacrene	0.57
Sabinene hydrate, cis	2.15	α-Farnesene	0.10
Terpinolene	1.32	δ-Cadinene	0.10
Linalool	1.97	Spathulenol	0.23
(Z)-*p*-menth-2-en-1-ol	0.72	Caryophyllene oxide	0.62
Camphor	0.28		
		Total identified	96.33

**Table 3:** Identification of the polyphenolic compounds in *T. baicalensis*.

No.	Identification	Rt	UV Max	[M-H] *m*/*z*	Fragmentation Ions	Total, mg kg^−1^
1	Quinic acid	4.120	240	191.02	111.01, 87.01, 85.03	23.7
2	Chlorogenic acid	18.83	250/325	335.09	191.06	44.6
3	Caffeic acid	20.64	322	179.03	135.04	78.8
4	Apigenin C-hexoside-C-hexoside	21.54	271/342	593.15	473.11	228.1
5	Rosmarinic acid derivative	22.73	282	377.09	359.08, 197.05, 161.02	136.1
6	5-O-feruloyquinic acid	24.36	325	367.10	191.06, 173.05	29.3
7	Quercetin-7-O-glucuronide	25.47	344	477.07	301.04	586.1
8	Luteolin-7-O-glucuronide	29.13	283/335	461.07	285.04, 113.02	10,112.1
9	Apigenin-7-O-glucuronide	33.26	269/342	445.08	269.05, 175.02, 113.02,	3634.4
10	Rosmarinic acid	34.30	253/329	359.08	197.05, 161.02	1840.4
					Total polyphenols: 16,713.6	

**Table 4:** Lipid components in the *T. baicalensis* extract.

Compound	Total, %
Caproic acid (C6:0)	0.89
Myristic acid (C14:0)	1.44
Pentadecanoic acid (C15:0)	0.61
Palmitic acid (C16:0)	31.05
Stearic acid (C18:0)	2.45
Arachidic acid (C20:0)	3.49
Behenic acid (C22:0)	1.30
Lignoceric acid (C24:0)	3.09
Nonanedioic acid, dimethyl-	3.97
Benzene-1,2-dicarboxylic acid, butyl-	1.07
Heptadecanoic acid, ethyl-	2.37
2-methyloctacosane	1.20
Pentacosane	1.51
Hexacosane	11.54
Heptacosane	1.51
Octacosane	1.98
Nonacosane	9.57
Arachidic acid	3.49
1,4-dihydroxy-p-menth-2-ene	1.68
Borneol	1.82
Ursolic acid	4.24
Oleanolic acid	9.72

**Table 5:** Minimal inhibitory concentrations of the ethanol extract of *T. baicalensi* determined by the microdilution method.

Gram+	MIC, %(*v*/*v*)	Gram-	MIC, %(*v*/*v*)	Fungi	MIC, %(*v*/*v*)
*M. flavus*	4.162	*P. fluorescens*	4.162	*S. cerevisiae*	0.260
*S. aureus*	16.667	*P. aeruginosa*	8.325	*C. vini*	2.081
*B. subtilis*	33.333	*E. coli*	8.325	*A. niger*	8.325
*S. epidermidis*	33.333	*E. aerogenes*	16.667	*P. expansum*	8.325

**Table 6:** Comparison of individual compounds in volatile oils of *Thymus* genus.

Species	Thymol (%)	Carvacrol (%)	*p*-Cymene (%)	γ-Terpinene (%)	Reference
*T. kotschyanus*	26.3–31.2	19.5–24.3	11.2–17.6	5.3–8.4	[27]
*T. musilii*	67.7	3.4	4.6	2.6	[28]
*T. daenensis*	47.08–82.01	0.77–24.39	2.76–5.37	1.06–4.07	[29]
*T. caramanicus*	4.14	65.52	13.21	4.44	[30]
*T. migricus*	1.41	0.29	-	-	[31]
*T. proximus*	0.05	8.47	44.26	33.17	[32]
*T. trautvetteri*	63.3–71.2	5.35–12.3	2.16–3.18	0.37–1.09	[33]
*T. fedtschenkoi*	50.61	6.58	7.69	3.16	[34]
*T. vulgaris*	3.99	56.79	12.8	11.17	[35]
*T. capitatus*	47.2–57.1	5.7–8.5	12.3–15.1	4.9–10.0	[36]
*T. zygis*	19.5	16.3	22.0	7.4	[37]

## Data Availability

The data presented in this study are available in this manuscript.

## Abbreviations

TFC	total flavonoid content
TPC	total phenolic content
GAE	gallic acid
QE	quercitin
DPPH	2,2-difenylo-1-pikrylohydrazyl
IC_50_	half-maximal inhibitory concentration
GC-MS	gas chromatography-mass spectroscopy
LC-MS	liquid chromatography-mass spectroscopy
MIC	minimum inhibitory concentration
TSFA	total saturated fatty acid
TMFA	total monounsaturated fatty acid
TPFA	total polyunsaturated fatty acid
